# Serum hepatitis B core antibody titer use in screening for significant fibrosis in treatment-naïve patients with chronic hepatitis B

**DOI:** 10.18632/oncotarget.14323

**Published:** 2016-12-28

**Authors:** Min-ran Li, Huan-wei Zheng, Jian-hua Lu, Shun-mao Ma, Li-hong Ye, Zhi-quan Liu, Hai-cong Zhang, Yun-yan Liu, Ying Lv, Yan Huang, Er-hei Dai, Dian-xing Sun

**Affiliations:** ^1^ Division of Liver Disease, The Fifth Hospital of Shijiazhuang, Hebei Medical University, Shijiazhuang, China; ^2^ Department of General Surgery, Huabei Petroleum General Hospital, Renqiu, China; ^3^ Department of Liver Diseases, Bethune International Peace Hospital, Shijiazhuang, China

**Keywords:** diagnostics, quantitative anti-HBc, chronic hepatitis B, liver fibrosis

## Abstract

**Background:**

Previous studies have revealed that hepatitis B core antibody (anti-HBc) levels vary throughout the different phases of treatment-naïve chronic hepatitis B (CHB) patients and can be used as a predictor of treatment response in both interferon-α and nucleoside analogue therapies. However, few data have been published regarding the relationship between quantitative anti-HBc (qAnti-HBc) levels and liver fibrosis in patients with CHB.

**Results:**

A total of 489 HBeAg-positive (HBeAg (+)) and 135 HBeAg-negative (HBeAg (−)) patients were recruited. In both HBeAg (+) and HBeAg (−) groups, the S0−1/S0 subjects had significantly lower qAnti-HBc levels than the S2−4 subjects (*p* < 0.05). Multiple logistic regression analysis showed that the parameters for predicting significant fibrosis (S ≥ 2) included age, PLT and qAnti-HBc. In HBeAg (+) subjects, the AUROC of qAnti-HBc for predicting significant fibrosis was 0.734 (95% CI 0.689 to 0.778) and the optimal cut-off was 4.58 log10IU/mL, with a sensitivity of 63.08% and a specificity of 74.83%. In HBeAg (−) subjects, the AUROC was 0.707 (95% CI 0.612 to 0.801) and the optimal cut-off value was 4.37 log10IU/mL, with a sensitivity of 75.53% and a specificity of 56.10%.

**Materials and Methods:**

From 2012 to 2015, we conducted a cross-sectional study of treatment-naïve CHB patients. Liver biochemistry, hepatitis B virus (HBV) serological markers, HBV DNA, hepatitis B surface antigen (HBsAg) titers and HBV genotype were determined using commercial assays, and serum qAnti-HBc levels were measured using double-sandwich immunoassay. Liver biopsies and serum samples were obtained on the same day.

**Conclusions:**

The present study showed an association between high serum qAnti-HBc levels and significant fibrosis (S ≥ 2) in treatment-naïve CHB patients. Furthermore, we described a serum qAnti-HBc cut-off for predicting significant fibrosis in CHB patients infected with HBV genotype B or C.

## INTRODUCTION

Worldwide, chronic hepatitis B virus (HBV) infection is a significant public health problem. The spectrum of disease and natural history of chronic HBV infection are highly variable, ranging from an inactive carrier status to progressive chronic hepatitis, which may lead to cirrhosis, liver failure and hepatocellular carcinoma (HCC) [1, 2]. Each year, 0.5–1.0 million people die from late stage chronic HBV infection-related liver disease [3]. Liver fibrosis, the natural wound healing process of liver necroinflammation, is an essential pathogenic process that leads to liver cirrhosis and disease progression [4, 5]. It is therefore critical to identify fibrosis in the early stage.

Liver biopsy is generally considered the reference standard for identifying liver fibrosis, as it allow for direct measurements. However, this procedure is invasive and associated with the risk of serious complications [6, 7]. Therefore, liver biopsy has limited applicability and does not allow dynamic observations of the liver fibrosis stage. The use of non-invasive techniques to assess liver fibrosis are gaining increasing interest. Currently, several non-invasive methods can predict liver fibrosis before treatment, such as the aspartate aminotransferase-platelet index (APRI) [8], the fibrosis index based on four factors (FIB-4) [9, 10] and transient elastography [11, 12]. However, most non-invasive tests have good diagnostic accuracy only in excluding advanced fibrosis or cirrhosis [13, 14].

Recently, an inverse correlation has been described between levels of the hepatitis B surface antigen (HBsAg) and the liver fibrosis stage in HBeAg-positive patients [15, 16]. As another classical serologic HBV marker, one study has suggested that the baseline levels of quantitative hepatitis B core antibody (qAnti-HBc) may serve as a novel biomarker for predicting treatment response in chronic hepatitis B (CHB) patients receiving interferon-α or nucleos(t)ide analogue therapy [17]. Other investigators have found that the levels of qAnti-HBc vary in different phases of treatment-naïve CHB patients and are highest in the immune clearance (IC) phase [18, 19]. These findings highlight the prognostic value of qAnti-HBc levels in CHB patients. However, the association between qAnti-HBc levels and liver fibrosis remains unspecified. Therefore, the aims of this study were to investigate the association between qAnti-HBc levels and histological fibrosis scores and to evaluate the use of quantitative biomarkers as tools to assess liver fibrosis in treatment-naive CHB patients.

## RESULTS

### Patient characteristics

**Figure 1:**
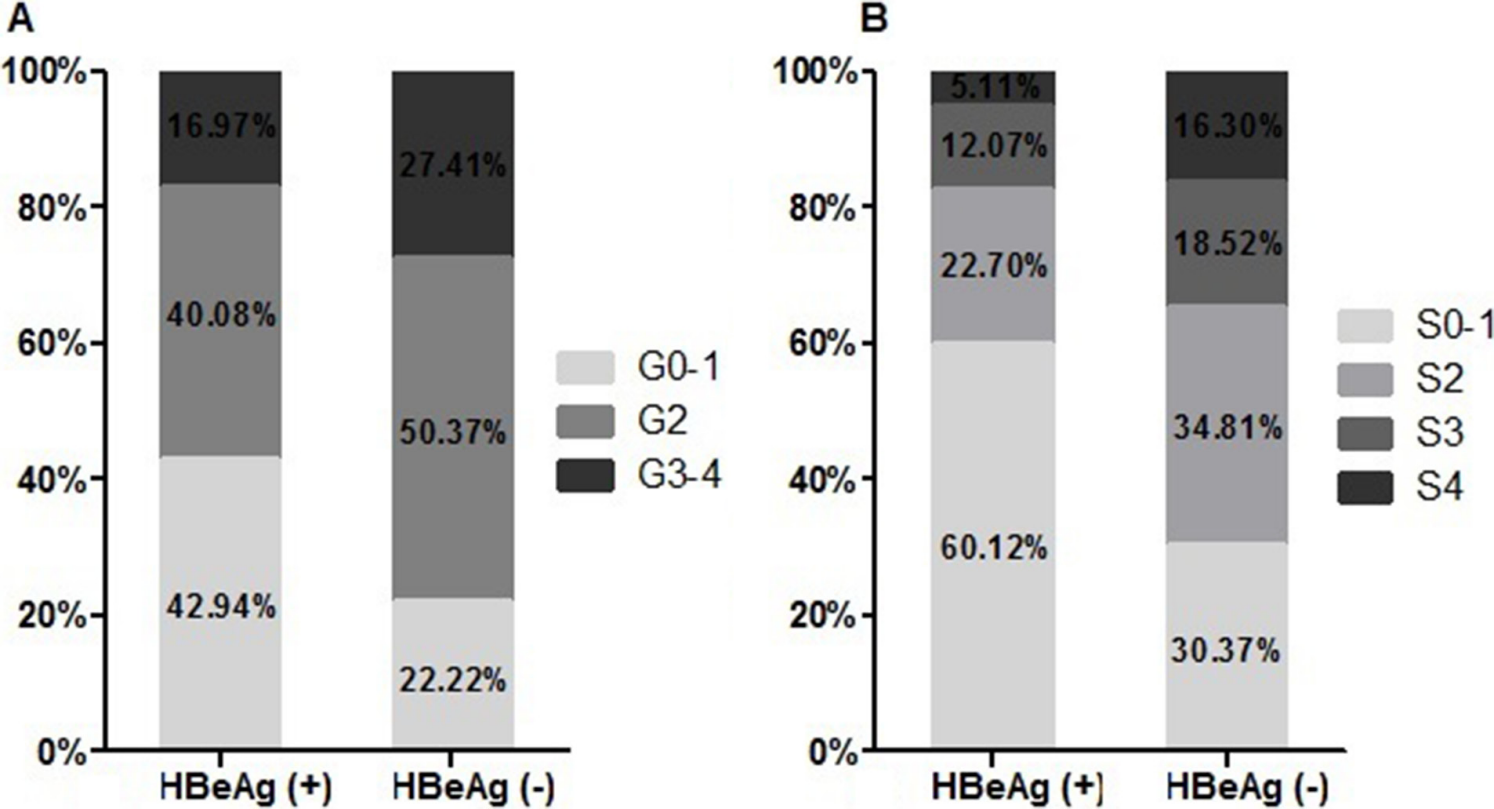
Distribution of liver necroinflammation grades **(A)** and fibrosis stages **(B)** by HBeAg status.

A total of 489 HBeAg-positive (HBeAg (+)) and 135 HBeAg-negative (HBeAg (−)) patients were included in the study. The baseline characteristics of the patients are presented in Table 1. There were more male than female patients in the study population. 68.59% of patients in both the HBeAg (+) and HBeAg (−) groups were male. The HBeAg (+) patients were younger than the HBeAg (−) patients (*p* < 0.001). Mean platelet (PLT) levels were significantly higher in HBeAg (+) patients. No significant differences were found for gender, ALT level, AST level, or HBV genotype between the two cohorts (all *p* > 0.05). The HBeAg (+) individuals exhibited significantly higher serum levels of both HBV DNA and HBsAg compared with HBeAg (−) patients. However, the HBeAg (+) group presented a significantly lower average qAnti-HBc level than the HBeAg (−) group. The distributions of the liver necroinflammation grades and fibrosis stages of the two cohorts are shown in Figure 1A and 1B. Among the HBeAg (+) patients, 294 patients (60.12%) had insignificant fibrosis (< S2), which was significantly higher than the proportion in the HBeAg (−) group (30.37%, *p* < 0.001). The proportion of patients with insignificant necroinflammation (< G2) among the two patient groups was also significantly different (42.94% and 22.22%, respectively, *p* = 0.003).

**Table 1:** Patient characteristics

	All (*n* = 624)	HBeAg (+) (*n* = 489)	HBeAg (−) (*n* = 135)	*p* value*
Gender, M/F	428/196	330/159	98/37	0.30
Age, years	32.79 ± 11.68	30.94 ± 10.59	39.51 ± 12.95	< 0.001
PLT, 10^9^/L	188.28 ± 61.00	195.27 ± 58.33	162.95 ± 63.87	< 0.001
ALT, U/L	75 (40–152)	72 (38–144)	99 (47–169)	0.15
AST, U/L	43 (26–85)	40 (25–80)	58 (34–97)	0.32
TBIL, μmol/L	19.80 ± 16.22	19.06 ± 15.12	22.51 ± 19.55	0.03
HBV DNA, log_10_IU/mL	6.69 ± 1.52	7.16 ± 1.18	4.99 ± 1.40	< 0.001
HBsAg, log_10_IU/mL	3.88 ± 0.71	4.00 ± 0.70	3.46 ± 0.57	< 0.001
qAnti-HBc, log_10_IU/mL	4.23 ± 0.99	4.14 ± 1.06	4.52 ± 0.61	< 0.001
HBV genotype, (%)**				0.23
B	53 (8.95)	45 (9.51)	8 (6.72)	
C	535 (90.37)	425 (89.85)	110 (92.44)	
D	3 (0.51)	3 (0.63)	0 (0.00)	
B/C	1 (0.17)	0 (0.00)	1 (0.84)	

* HBeAg (+) vs. HBeAg (−).

** 32 patients could not be genotyped with our assay.

### Association between histological fibrosis stage and qAnti-HBc levels

**Figure 2:**
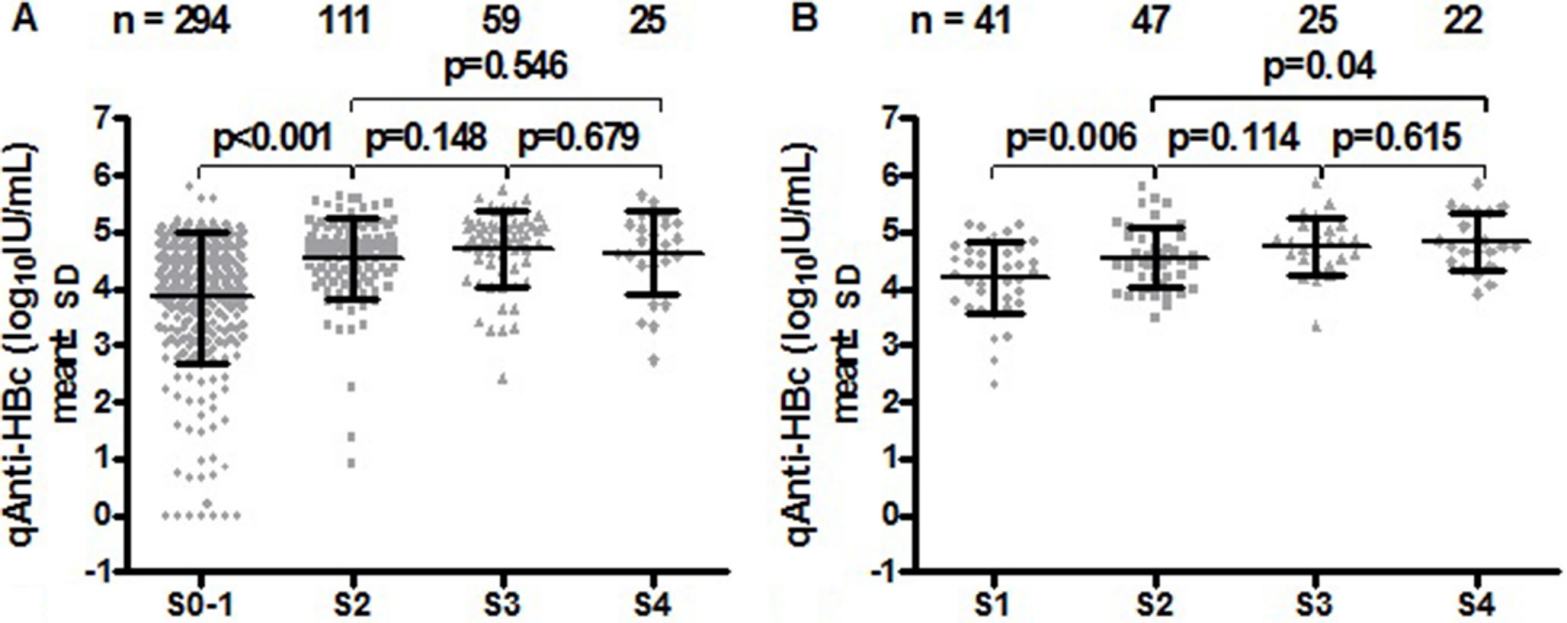
Correlation between serum qAnti-HBc levels and liver fibrosis stages in HBeAg (+) **(A)** and HBeAg (–) CHB patients **(B)**.

Among the HBeAg (+) CHB patients, the mean levels of qAnti-HBc for different stages of fibrosis were as follows: S0–1 (3.84 ± 1.14 log_10_IU/mL), S2 (4.54 ± 0.73 log_10_IU/mL), S3 (4.70 ± 0.68 log_10_IU/mL) and S4 (4.63 ± 0.74 log_10_IU/mL). The mean qAnti-HBc level of the S0–1 subjects was significantly lower than that of the S2, S3 and S4 subjects (*p* < 0.05) (Figure 2A). Among the HBeAg (−) patients, the mean levels of qAnti-HBc for the different stages of fibrosis were as follows: S1 (4.19 ± 0.64 log_10_IU/mL), S2 (4.55 ± 0.53 log_10_IU/mL), S3 (4.76 ± 0.51 log_10_IU/mL), and S4 (4.83 ± 0.50 log_10_IU/ mL). The mean qAnti-HBc levels in the S1 subjects were also significantly lower than those in the S2, S3, and S4 subjects (*p* < 0.05) (Figure 2B).

### Use of qAnti-HBc levels and other factors to distinguish significant fibrosis

When the presence or absence of significant fibrosis (S ≥ 2) was considered as a binary dependent variable in patients, univariable analyses indicated that age, PLT, ALT, AST, TB, HBV DNA, HBsAg and qAnti-HBc levels were associated with significant fibrosis in HBeAg (+) patients, whereas multiple logistic regression analysis identified that age, PLT and qAnti-HBc levels were associated with significant fibrosis (Table 2).

**Table 2:** Multiple logistic regression analysis of factors associated with significant fibrosis in HBeAg (+) patients

Parameter	Not significant	Significant	*P*	Multivariate			
				OR	95% CI	Wald	*P*
Age, years	27.72 ± 8.83	35.79 ± 11.19	< 0.001	1.07	1.04–1.09	28.10	**< 0.001**
PLT, 10^9^/L	211.00 ± 51.48	171.56 ± 60.14	< 0.001	0.99	0.99–1.00	17.60	**< 0.001**
ALT, U/L	76.00 ± 94.29	292.45 ± 386.03	< 0.001	1.00	1.00–1.01	2.24	0.135
AST, U/L	43.82 ± 51.58	186.44 ± 282.54	< 0.001	1.01	1.00–1.01	3.43	0.064
TBIL, μmol/L	16.27 ± 9.29	23.26 ± 20.37	< 0.001	1.01	0.98–1.03	0.15	0.696
HBV DNA, log_10_IU/mL	7.42 ± 1.05	6.77 ± 1.26	< 0.001	0.83	0.66–1.04	2.64	0.104
HBsAg, log_10_IU/mL	4.16 ± 0.70	3.77 ± 0.63	< 0.001	0.72	0.49–1.04	3.03	0.082
qAnti-HBc, log_10_IU/mL	3.84 ± 1.14	4.60 ± 0.72	< 0.001	1.77	1.28–2.45	11.85	**0.001**

In HBeAg (−) patients, univariable analyses indicated that only age, PLT, AST, HBV DNA and qAnti-HBc levels were associated with significant fibrosis. Subsequent multiple logistic regression analysis identified that PLT and qAnti-HBc levels were associated with significant fibrosis, which is similar to the results found for HBeAg (+) patients (Table 3).

**Table 3:** Multiple logistic regression analysis of factors associated with significant fibrosis in HBeAg (−) patients

Parameter	Not significant	Significant	*P*	Multivariate			
				OR	95% CI	Wald	*P*
Age, years	33.56 ± 12.73	42.11 ± 12.22	< 0.001	1.04	1.00–1.08	3.53	0.060
PLT, 10^9^/L	199.66 ± 52.90	146.94 ± 61.81	< 0.001	0.99	0.98–1.00	11.00	**0.001**
ALT, U/L	112.68 ± 253.23	190.89 ± 209.30	0.064	1.00	1.00–1.00	0.60	0.441
AST, U/L	60.95 ± 114.17	115.44 ± 146.46	0.036	1.01	1.00–1.02	2.30	0.129
TBIL, μmol/L	19.85 ± 19.52	23.67 ± 19.56	0.298	0.99	0.94–1.04	0.23	0.633
HBV DNA, log_10_IU/mL	4.51 ± 1.52	5.20 ± 1.30	0.008	1.32	0.94–1.85	2.64	0.104
HBsAg, log_10_IU/mL	3.54 ± 0.67	3.43 ± 0.53	0.308	1.04	0.48–2.26	0.01	0.926
qAnti-HBc, log_10_IU/mL	4.19 ± 0.64	4.67 ± 0.53	< 0.001	3.36	1.33–8.47	6.59	**0.010**

**Figure 3:**
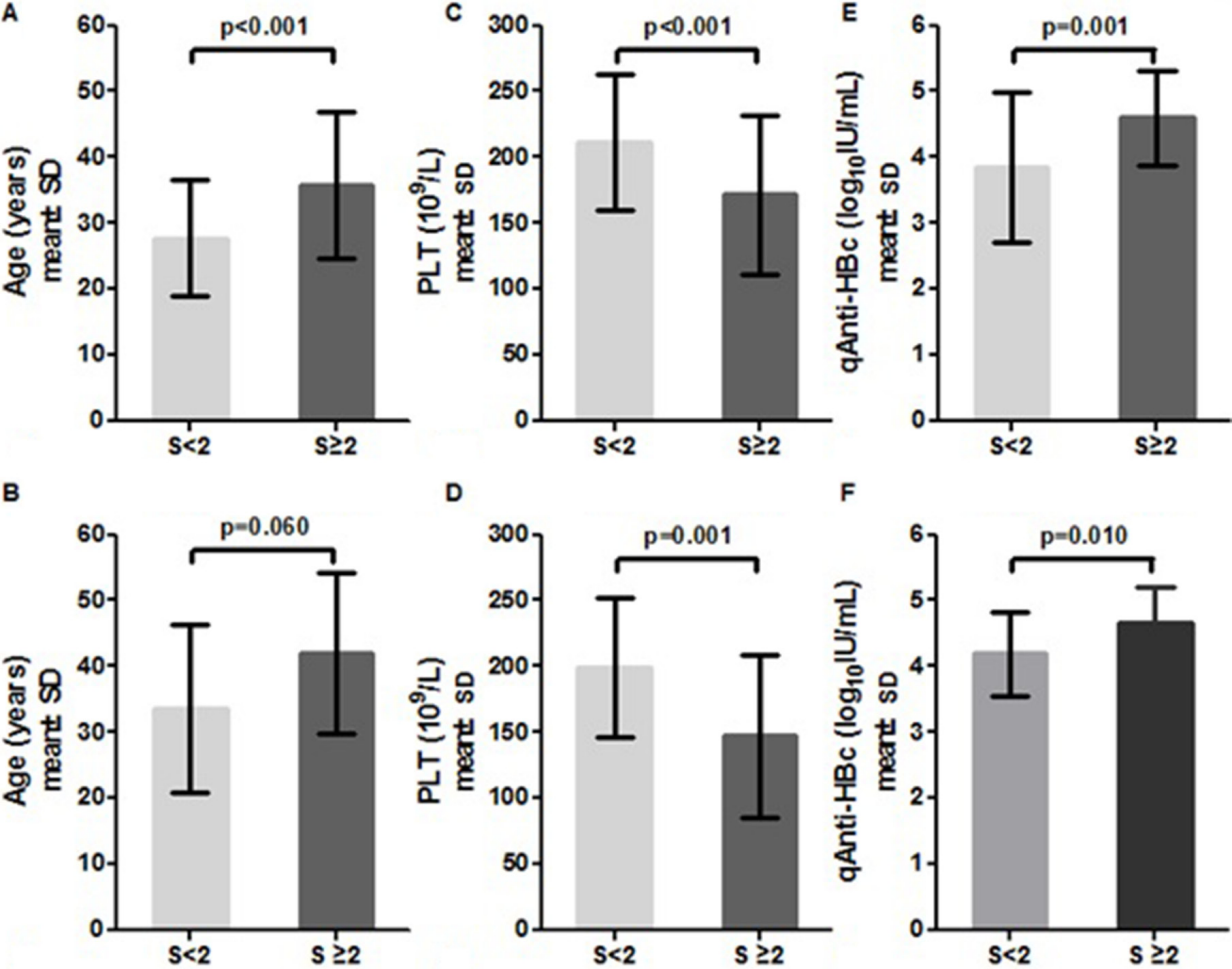
Distribution of age, PLT and qAnti-HBc levels in HBeAg (+) **(A, C, E)** and HBeAg (–) patients **(B, D, F)** stratified by fibrosis stage.

In both the HBeAg (+) and HBeAg (−) groups, patients with significant fibrosis exhibited significantly lower PLT levels compared to patients with insignificant fibrosis. However, mean serum qAnti-HBc levels were significantly higher in patients with significant fibrosis. Patients with significant fibrosis were older than patients with insignificant fibrosis (Figure 3).

### AUROC of qAnti-HBc associated with significant fibrosis

**Figure 4:**
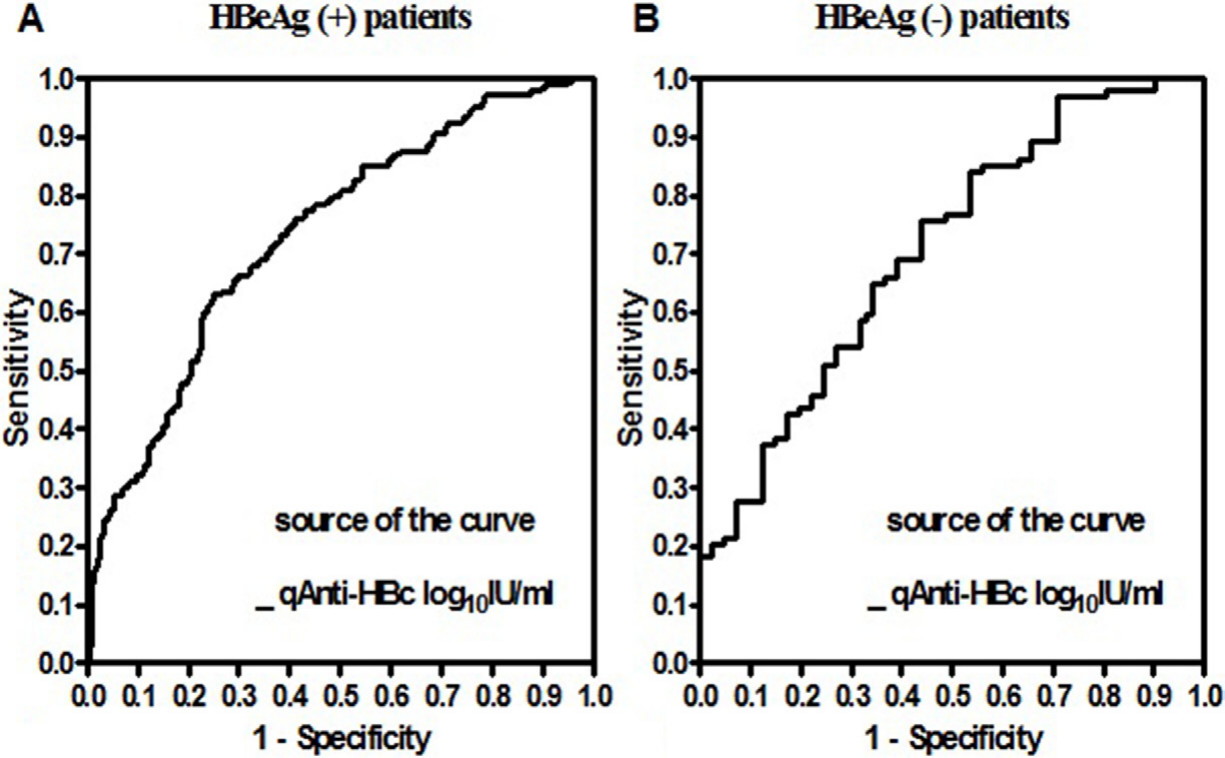
Receiver operating characteristic curves of serum qAnti-HBc levels used to distinguish moderate to severe fibrosis in HBeAg (+) **(A)** and HBeAg (–) **(B)** CHB patients.

When an ROC analysis was performed to discriminate significant fibrosis on the basis of serum qAnti-HBc levels in HBsAg (+) patients, the AUROC was 0.734 (95% CI 0.689 to 0.778), and the optimal cut-off was 4.58 log_10_IU/mL, with a sensitivity of 63.08% and a specificity of 74.83%. The AUROC of serum qAnti-HBc levels was similar to that of age (0.729, 95% CI 0.683 to 0.776) and PLT (0.703, 95% CI 0.654 to 0.752), but it was lower than the AUROC values for APRI (0.847, 95% CI 0.810 to 0.884) and FIB-4 (0.836, 95% CI 0.797 to 0.875) (Table 4, Figure 4A).

**Table 4:** Diagnostic performances of age, PLT, qAnti-HBc, APRI and Fib-4 in HBeAg (+) and HBeAg (−) patients

Group	Baseline value	AUROC	95% CI	Cut-off	Sensitivity	Specificity	PPV	NPV
HBeAg (+)	Age, years	0.729	0.683–0.776	32.50	55.38%	80.95%	65.85%	73.23%
	PLT, 10^9^/L	0.703	0.654–0.752	176.50	60.51%	74.49%	61.14%	73.99%
	qAnti-HBc, log_10_IU/mL	0.734	0.689–0.778	4.58	63.08%	74.83%	62.44%	75.34%
	APRI	0.847	0.810–0.884	0.25	81.03%	77.89%	70.85%	86.09%
	FIB-4	0.836	0.797–0.875	1.30	65.13%	93.54%	86.99%	80.17%
HBeAg (−)	PLT, 10^9^/L	0.761	0.678–0.843	162.5	65.96%	82.93%	89.86%	51.52%
	qAnti-HBc, log_10_IU/mL	0.707	0.612–0.801	4.37	75.53%	56.10%	79.78%	50.00%
	APRI	0.824	0.750–0.899	0.43	64.89%	87.80%	92.42%	52.17%
	FIB-4	0.821	0.742–0.900	1.33	76.60%	82.93%	91.14%	60.71%

In HBeAg (−) patients, the AUROC of serum qAnti-HBc levels for the prediction of significant fibrosis (0.707, 95% CI 0.612 to 0.801) was also similar to that of PLT (0.761, 95% CI 0.678 to 0.843), and it was lower than that of APRI (0.824, 95% CI 0.750 to 0.899) and FIB-4 (0.821, 95% CI 0.742 to 0.900). For predicting significant fibrosis, the optimal cut-off value of qAnti-HBc was 4.37 log_10_IU/mL, with a sensitivity of 75.53% and a specificity of 56.10%. (Table 4, Figure 4B).

## DISCUSSION

Liver fibrosis, which is the result of the normal wound healing process of necroinflammation frequently caused by chronic HBV infection, is essential to the pathogenic processes that lead to cirrhosis. In terms of establishing cirrhosis, the annual incidence of HBV-related HCC in CHB patients ranges from 2% to 5%. The 2012 European Association for the Study of the Liver (EASL) clinical practice guideline recommended that patients should be considered for treatment when they have at least moderate fibrosis (S ≥ 2) [20]. Therefore, the ability to identify CHB patients with moderate to severe fibrosis (S2–4), with a high risk of disease progression, has clear clinical significance.

Anti-HBc, as classical serological marker of HBV infection, is an indicator of both past and persistent HBV infection. The present study provided the first detailed investigation of the relationship between serum qAnti-HBc levels and liver fibrosis stages across a large cohort of treatment-naïve patients with CHB. All patients’ serum samples were taken on the day of their biopsies and therefore represent an accurate snapshot of the state of disease situation at that specific time. We observed a correlation between qAnti-HBc levels and liver fibrosis. The qAnti-HBc levels were positively correlated with stage of fibrosis in treatment-naïve CHB patients. Both HBeAg (+) and HBeAg (−) patients with moderate to severe fibrosis (S2–S4) showed significantly higher levels of qAnti-HBc compared with patients with no or mild fibrosis (S0-S1/S1). No significant differences were found in the qAnti-HBc titers among the S2-S4 subjects, excepted between S2 and S4 subjects in HBeAg (−) group.

Most previous studies have investigated the predictors for liver fibrosis that can avoid liver biopsy in CHB patients, especially for patients with moderate to severe fibrosis, who required immediate antiviral therapy. In the present study, we examined routine parameters for predicting moderate to severe liver fibrosis. Our study showed that PLT and qAnti-HBc were independently associated with significant fibrosis in different chronic HBV groups, that is, HBeAg (+) and HBeAg (−) patients. And age was also independently associated with significant fibrosis in HBeAg (+) group. Age and PLT had been shown in previous studies to be associated with significant fibrosis in CHB [21–24]. Age was an important predictor because the progression of fibrosis is time-dependent in CHB. Low platelet count was associated with advanced liver fibrosis through the altered production of thrombopoietin, and this occurred independent of demographic and biochemical characteristics, hepatic necroinflammatory activity, portal hypertension and splenomegaly [25]. Our analyses found that the AUROC values of serum qAnti-HBc level for significant fibrosis were 0.734 and 0.707 in HBeAg (+) and HBeAg (−) patients, respectively, which similar to that of age (0.729) and PLT (0.703 and 0.761). APRI (0.847 and 0.824) and FIB-4 (0.836 and 0.821) tended to present more accurate results for identification of significant fibrosis than qAnti-HBc. However, APRI consists of PLT and AST. In addition to these two factors, FIB-4 includes age and ALT.

The present study demonstrated that serum qAnti-HBc levels can play an important role in identifying treatment-naïve CHB patients with significant fibrosis and may help reduce the need for liver biopsies. To our knowledge, our study was the first to formally use liver biopsy as an outcome measure to assess the role of qAnti-HBc titers in distinguishing significant fibrosis. The mechanistic explanation for why higher serum qAnti-HBc levels are associated with increasingly severe liver fibrosis in treatment-naïve CHB patients is unclear. qAnti-HBc levels in immune clearance and HBeAg negative hepatitis phases were significantly higher than those in the immune tolerance and low or no-replicative phases [18, 19]. Moreover, qAnti-HBc levels were positively associated with ALT and liver inflammation grades [26]. These results suggested that qAnti-HBc might be a surrogate parameter indicating ongoing anti-HBV immune activation. Liver fibrosis results from the sustained wound healing response of the liver to chronic injury. Liver injury observed during chronic HBV infections appears to be primarily caused by the host's anti-HBV immune activation. Both the innate and adaptive immune systems are pivotal in the fibrotic cascade. Perhaps immune activation is the link between qAnti-HBc levels and liver fibrosis.

In conclusion, our data suggest that treatment-naïve CHB patients with moderate to severe fibrosis (S ≥ 2) had a significant higher level of qAnti-HBc compared with patients with no or mild fibrosis (S < 2). And our analyses established an optimal cutoff value of qAnti-HBc for distinguishing HBeAg (+) (> 4.58 log_10_IU/mL) and HBeAg (−) (> 4.37 log_10_IU/mL) patients with significant fibrosis (S ≥ 2). The use of this prediction score of qAnti-HBc may potentially reduce the need for liver biopsies and help guide clinical decision making in the management of HBV genotype B or C associated CHB patients.

## MATERIALS AND METHODS

### Patients

This was a cross-sectional study consisting of patients enrolled at the Division of Liver Disease, The Fifth Hospital of Shijiazhuang, Hebei Medical University between January 2012 and December 2015. All patients were HBsAg-positive for at least six months before study entry. Other inclusion criteria were as follows: treatment-naïve and availability of relevant patient laboratory and clinical data. The exclusion criteria were as follows: (1) hepatitis A, C, or E or human immunodeficiency virus co-infection; (2) decompensated liver cirrhosis or HCC; (3) treatment with antiviral therapy or any liver functional protection therapy to alleviate the hepatic inflammation; (4) immunosuppressive treatment; and (5) causes of liver disease other than HBV.

The study protocol was in compliance with the 1975 Declaration of Helsinki and was approved by the Medical Ethics Committee of The Fifth Hospital of Shijiazhuang. Written informed consent was obtained from all patients.

### Biochemical and serological tests

The serum samples used for measurements were obtained on the day of the biopsy. The complete blood cell counts and biochemical tests were performed using automated techniques. The upper limit of normal alanine transaminase (ALT) was 40 U/L. Hepatitis B e antigen (HBeAg) and antibodies to HBeAg (anti-HBe) were measured using commercially available immunoassays (Roche Diagnostics, Branchburg, NJ, USA). The serum HBV DNA levels were determined by real time fluorescence quantitative polymerase chain reaction (FQ-PCR) on an ABI 7500, with a lower detection limit of 500 IU/ml.

Serum HBsAg levels were quantified using an Elecsys HBsAg II quant assay (Roche Diagnostics, Branchburg, NJ, USA), with a diagnostic range from 0.05 to 130 IU/mL. Serial 1:10 dilutions were performed if the HBsAg levels were >130 IU/mL, according to the manufacturer's instructions. The serum qAnti-HBc level was measured using a newly developed double-sandwich immunoassay (Wantai, Beijing, China) that was calibrated using the WHO standard (NIBSC, UK) [27].

### Genotype determination by multiplex PCR

HBV genotypes were determined by analysis of the sequences obtained after amplification of the preS/S gene (approximately 1,410 bp spanning nucleotide positions 2825 to 1019).

### Liver biopsy

Percutaneous liver biopsies were performed using a 16-gauge needle with a biopsy length of at least 1.5 cm and including six or more portal tracts. The samples were formalin-fixed, paraffin-embedded, and prepared by hematoxylin-eosin staining for morphological evaluation, Masson's trichrome staining, and reticulin staining for fibrosis assessment. Liver biopsy specimens were scored for fibrosis according to the Scheuer classification: no fibrosis (S0); portal fibrosis (S1); septum formation (S2); architectural distortion (S3); cirrhosis, probable or definite (S4) [28]. “Insignificant fibrosis” was defined as a Scheuer fibrosis score equal to or less than 1. “Significant fibrosis” was defined as a Scheuer score more than or equal to 2.

### Statistical analysis

Categorical variables are expressed as counts and percentages and were analyzed using the χ^2^ or Fisher's exact test. Continuous variables are presented as the mean ± SD or the medians. Student's *t* test or the Mann-Whitney *U*-test was used for statistical comparisons where appropriate. Multinomial (binary) logistic regression was applied to evaluate factors predicting significant fibrosis based on histology. Receiver operating characteristic (ROC) curves and areas under the ROC curves (AUROC) were calculated to evaluate the diagnostic accuracy of qAnti-HBc for liver fibrosis activity. *P* values of < 0.05 were considered statistically significant. Statistical analyses were performed using SPSS ver. 19.0 software (SPSS, Chicago, IL, USA).

## Abbreviations

Anti-HBc: hepatitis B core antibody
CHB: chronic hepatitis B
qAnti-HBc: quantitative hepatitis B core antibody
HBV: hepatitis B virus
HBsAg: hepatitis B surface antigen
HBeAg: Hepatitis B e antigen
APRI: aspartate aminotransferase-platelet index
FIB-4: the fibrosis index based on four factors
ALT: alanine transaminase
PLT: platelet

## References

[1] McMahon BJ (2009). The natural history of chronic hepatitis B virus infection. Hepatology.

[2] Fattovich G (2003). Natural history and prognosis of hepatitis B. Semin Liver Dis.

[3] Liaw Y-F, Chu CM (2009). Hepatitis B virus infection. Lancet.

[4] Chan HL, Wong GL, Wong VW (2009). A review of the natural history of chronic hepatitis B in the era of transient elastography. Antivir Ther.

[5] Chu CM (2000). Natural history of chronic hepatitis B virus infection in adults with emphasis on the occurrence of cirrhosis and hepatocellular carcinoma. J Gastroenterol Hepatol.

[6] McGill DB, Rakela J, Zinsmeister AR, Ott BJ (1990). A 21-year experience with major hemorrhage after percutaneous liver biopsy. Gastroenterol.

[7] B Al Knawy, Shiffman M (2007). Percutaneous liver biopsy in clinical practice. Liver Int.

[8] Wai CT, Greenson JK, Fontana RJ, Kalbfleisch JD, Marrero JA, Conjeevaram HS, Lok AS (2003). A simple noninvasive index can predict both significant fibrosis and cirrhosis in patients with chronic hepatitis C. Hepatology.

[9] RB1 Thandassery, S Al Kaabi, Soofi ME, Mohiuddin SA, John AK, M Al Mohannadi, K Al Ejji, Yakoob R, Derbala MF, Wani H, Sharma M, N Al Dweik, Butt MT (2016). Mean Platelet Volume, Red Cell Distribution Width to Platelet Count Ratio, Globulin Platelet Index, and 16 Other Indirect Noninvasive Fibrosis Scores: How Much Do Routine Blood Tests Tell About Liver Fibrosis in Chronic Hepatitis C?. J Clin Gastroenterol.

[10] Sterling RK, Lissen E, Clumeck N, Sola R, Correa MC, Montaner J, M S Sulkowski, Torriani FJ, Dieterich DT, Thomas DL, Messinger D, M; Nelson, APRICOT Clinical Investigators (2006). Development of a simple noninvasive index to predict significant fibrosis in patients with HIV/HCV coinfection. Hepatology.

[11] Castéra L, Bernard PH, Le Bail B, Foucher J, Trimoulet P, Merrouche W, Couzigou P, de Lédinghen V (2011). Transient elastography and biomarkers for liver fibrosis assessment and follow-up of inactive hepatitis B carriers. Aliment Pharmacol Ther.

[12] Kim SU, Kim BK, Park JY, Y Kim do, Ahn SH, Song K, Han KH (2016). Transient Elastography is Superior to FIB-4 in Assessing the Risk of Hepatocellular Carcinoma in Patients With Chronic Hepatitis B. Medicine Baltimore.

[13] WHO (2015). Guidelines for the Prevention, Care and Treatment of Persons with Chronic Hepatitis B Infection.

[14] Xiao G, Yang J, Yan L (2015). Comparison of diagnostic accuracy of aspartate aminotransferase to platelet ratio index and fibrosis-4 index for detecting liver fibrosis in adult patients with chronic hepatitis B virus infection: a systemic review and meta-analysis. Hepatology.

[15] Martinot-Peignoux M, Carvalho-Filho R, Lapalus M, Netto-Cardoso AC, Lada O, Batrla R, Krause F, Asselah T, Marcellin P (2013). Hepatitis B surface antigen serum level is associated with fibrosis severity in treatment-naïve, e antigen-positive patients. J Hepatol.

[16] Seto WK, Wong DK, Fung J, Ip PP, Yuen JC, Hung IF, Lai CL, Yuen MF (2012). High hepatitis B surface antigen levels predict insignificant fibrosis in hepatitis B e antigen positive chronic hepatitis B. Plos One.

[17] Yuan Q, Song LW, Liu CJ, Li Z, Liu PG, Huang CH, Yan Y, Ge SX, Wang YB, Peng CY, Zhang J, Kao JH, Chen DS (2013). Quantitative hepatitis B core antibody level may help predict treatment response in chronic hepatitis B patients. Gut.

[18] Jia W, Song LW, Fang YQ, Wu XF, Liu DY, Xu C, Wang XM, Wang W, Lv DX, Li J, Deng YQ, Wang Y, Huo N (2014). Antibody to hepatitis B core antigen levels in the natural history of chronic hepatitis B: a prospective observational study. Medicine.

[19] Song LW, Liu PG, Liu CJ, Zhang TY, Cheng XD, Wu HL, Yang HC, Hao XK, Yuan Q, Zhang J, Kao JH, Chen DS, Chen PJ (2015). Quantitative hepatitis B core antibody levels in the natural history of hepatitis B virus infection. Clin Microbiol Infect.

[20] (2012). European Association for the Study of the Liver. EASL clinical practice guidelines: management of chronic hepatitis B virus infection. J Hepatol.

[21] Zeng MD, Lu LG, Mao YM, Qiu DK, Li JQ, Wan MB, Chen CW, Wang JY, Cai X, Gao CF, Zhou XQ (2005). Prediction of significant fibrosis in HBeAg-positive patients with chronic hepatitis B by anoninvasive model. Hepatology.

[22] Hui AY, Chan HL, Wong VW, Liew CT, Chim AM, Chan FK, Sung JJ (2005). Identification of chronic hepatitis B patients without significant liver fibrosis by a simplenoninvasive predictive model. Am J Gastroenterol.

[23] Tan Y, Ye Y, Zhou X, Chen L, Wen D (2015). Age as a predictor of significant fibrosis features in HBeAg-negative chronic hepatitis B virus infection with persistently normal alanine aminotransferase. PLoS One.

[24] Wang Y, Xu MY, Zheng RD, Xian JC, Xu HT, Shi JP, Li SB, Qu Y, Dong YW, Lu LG (2013). Prediction of significant fibrosis and cirrhosis in hepatitis B e-antigen negative patients with chronichepatitis B using routine parameters. Hepatol Res.

[25] Adinolfi LE, Giordano MG, Andreana A, Tripodi MF, Utili R, Cesaro G, Ragone E, E Durante Mangoni, Ruggiero G (2001). Hepatic fibrosis plays a central role in the pathogenesis of thrombocytopenia in patients with chronic viralhepatitis. Br J Haematol.

[26] Li MR, Lu JH, Ye LH, Sun XL, Zheng YH, Liu ZQ, Zhang HC, Liu YY, Lv Y, Huang Y, Dai EH (2016). Quantitative hepatitis B core antibody level is associated with inflammatory activity in treatment-naïve chronic hepatitis B patients. Medicine (Baltimore).

[27] Li A, Yuan Q, Huang Z, Fan J, Guo R, Lou B, Zheng Q, Ge S, Chen Y, Su Z, Yeo AE, Chen Y, Zhang J (2010). Novel double-antigen sandwich immunoassay for human hepatitis B core antibody. Clin Vaccine Immunol.

[28] Scheuer PJ, Standish RA, Dhillon AP (2002). Scoring of chronic hepatitis. Clinics In Liver Disease.

